# IDEA: Interactive Display for Evolutionary Analyses

**DOI:** 10.1186/1471-2105-9-524

**Published:** 2008-12-08

**Authors:** Rain Simons, Anup Mahurkar, Jonathan Crabtree, Jonathan H Badger, Jane M Carlton, Joana C Silva

**Affiliations:** 1 Institute for Genome Sciences, University of Maryland School of Medicine, Baltimore, MD 21201, USA; 2 The J. Craig Venter Institute, San Diego, CA 92121, USA; 3 Department of Medical Parasitology, New York University School of Medicine, New York, NY 10010, USA; 4 Department of Microbiology and Immunology, University of Maryland School of Medicine, Baltimore, MD 21201, USA

## Abstract

**Background:**

The availability of complete genomic sequences for hundreds of organisms promises to make obtaining genome-wide estimates of substitution rates, selective constraints and other molecular evolution variables of interest an increasingly important approach to addressing broad evolutionary questions. Two of the programs most widely used for this purpose are *codeml *and *baseml*, parts of the PAML (Phylogenetic Analysis by Maximum Likelihood) suite. A significant drawback of these programs is their lack of a graphical user interface, which can limit their user base and considerably reduce their efficiency.

**Results:**

We have developed IDEA (Interactive Display for Evolutionary Analyses), an intuitive graphical input and output interface which interacts with PHYLIP for phylogeny reconstruction and with *codeml *and *baseml *for molecular evolution analyses. IDEA's graphical input and visualization interfaces eliminate the need to edit and parse text input and output files, reducing the likelihood of errors and improving processing time. Further, its interactive output display gives the user immediate access to results. Finally, IDEA can process data in parallel on a local machine or computing grid, allowing genome-wide analyses to be completed quickly.

**Conclusion:**

IDEA provides a graphical user interface that allows the user to follow a *codeml *or *baseml *analysis from parameter input through to the exploration of results. Novel options streamline the analysis process, and post-analysis visualization of phylogenies, evolutionary rates and selective constraint along protein sequences simplifies the interpretation of results. The integration of these functions into a single tool eliminates the need for lengthy data handling and parsing, significantly expediting access to global patterns in the data.

## Background

The estimation of substitution rates and selective constraints is an essential step in the study of fundamental questions in evolutionary biology, including the evolution and organization of genomes [1-3], and the genetic basis of species differences [4-6]. Estimation approaches based on maximum-likelihood (ML) provide highly accurate estimates of substitution rates and related variables of interest [7,8]. Arguably the most popular ML-based program for evaluating evolutionary hypotheses is PAML (Phylogenetic Analysis by Maximum Likelihood), and the two programs *codeml *and *baseml *are its core use cases [9]. *codeml *in particular allows the user to obtain estimates of substitution rates per branch and/or per site and to compare the likelihood of multiple models of molecular evolution given the data and a phylogenetic tree.

The strong mathematical capabilities of *codeml *and *baseml *are counterbalanced, however, by their lack of a graphical user interface (GUI). The availability of the complete genomic sequences of many organisms and the consequent increase in popularity of genomic-scale evolutionary studies have made addressing this deficit a priority. A program that requires the laborious creation of text-based input files does not scale well to an analysis of hundreds of genes. The benefits of using a set of loci representative of an entire genome to better elucidate the properties of species or clades or the evolution of genome properties are limited when there is no easy way to compare the results obtained for different genes.

We created IDEA with the goal of easing the researcher's burden in two key areas. First, a graphical interface allows the easy input of parameters for many datasets, automating the distribution of jobs in parallel and allowing the user to monitor the progress of an analysis while it is executing. This input GUI also prevents initiation errors since the use of format-controlled and validated input fields is less error-prone than the editing of flat files. Second, the availability of a visual display of the main results immediately after the completion of an analysis eliminates the need for extensive handling and parsing of output files before the general attributes of the data can be accessed, and the interactive nature of the output GUI facilitates in-depth investigation.

## Implementation

IDEA is written mostly in Java; its design reflects the key consideration of extensibility. In particular, the incorporation of new parameters that may be added to future versions of PAML should not necessitate any modifications to the IDEA code. Perl is used as a scripting language to provide text input to third-party C programs such as *codeml*, *baseml *and the programs in the PHYLIP suite [10] and to parse their output. PAML and PHYLIP are not packaged with IDEA and must be installed prior to the installation of IDEA.

IDEA's graphical user interface is built from Swing components, and Java2D is used to create adjustable histograms. The visualization component that displays the distribution of selective constraints across a protein is written in Perl and makes use of the Apache Batik rasterizer [11] to convert scalable vector graphics (SVG) files to JPEG images. The phylogenetic-tree visualization component is written in JRuby [12], a Java implementation of Ruby that offers a 2x-5x performance advantage over traditional Ruby and integrates easily with Java. To avoid unnecessary computation, both visualization components create images on demand the first time the user requests them and then save them for later reuse.

The distribution of computing jobs is handled by the Workflow process manager, a multi-threaded Java application that manages the creation, execution and monitoring of a directed acyclic graph (DAG) of commands. Workflow is ideally suited to bioinformatics applications, which often involve computational pipelines hierarchically composed of many analysis components. Execution of jobs on the grid is accomplished by interaction with any of multiple distributed resource managers (DRMs) the user may have available; although the current version of Workflow has been tested with only Condor and Sun Grid Engine, the upcoming version of Workflow, expected later in 2008, will support other DRMs through the Distributed Resource Management Application API (DRMAA). Workflow is included with IDEA and is also available for download separately at http://sourceforge.net/projects/tigr-workflow/. Supported platforms include Linux, Solaris, Mac OS X and Tru64.

## Results

### Data input and processing

**Figure 1:**
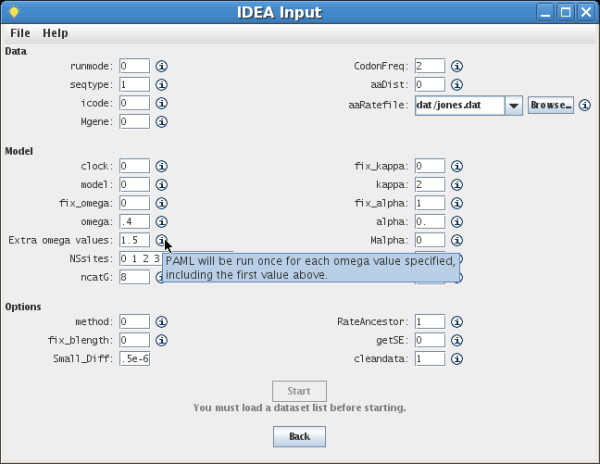
**IDEA input GUI**. IDEA's parameter input interface. Parameters for *codeml *or *baseml *are organized in three categories: data, model and other options. Mousing over the information icon next to a parameter brings up an explanation of that parameter (blue text box).

An IDEA user first elects to run either *codeml*, which performs maximum likelihood analysis using codon substitution models, or *baseml*, which uses nucleotide substitution models. IDEA presents the parameters for the chosen PAML program grouped visually into three categories (data, model and other options) and allows the user to provide a value for each (Figure 1). Sensible default values are provided; the user may also load or save customized default values. A key to the interpretation of each parameter can be viewed by mousing over an icon next to that parameter's name (Figure 1). IDEA performs limited input validation to detect some common errors. A help menu connects the user to IDEA's online documentation.

IDEA augments the standard PAML parameters with additional options. As an alternative to PAML's existing support for multiple-locus analyses, IDEA's multi-dataset option offers the key advantages of increased speed and capacity: up to hundreds of genes may be analyzed simultaneously on a computing grid, and the total number of datasets is for practical purposes unlimited, making genome-wide analyses possible. Because *codeml*'s maximum likelihood estimation of ω (*d*_*N*_/*d*_*S*_) is an iterative algorithm that may arrive at a local likelihood optimum, another option allows the user to define multiple starting ω values. IDEA runs *codeml *once with each value and automatically displays only the results of the run in which the best likelihood score was obtained.

Because many PAML analyses require phylogenetic trees as input, PHYLIP [10] is integrated into the IDEA pipeline, allowing the user to quickly estimate phylogenetic trees as an initial step in analysis using maximum parsimony or neighbor joining (NJ). Maximum parsimony is used for datasets with six or fewer sequences, as implemented in *dnapenny *(exhaustive search using a branch-and-bound algorithm), while NJ (*dnadist *and *neighbor*) is used for larger datasets. These options are hard-coded in IDEA, and the programs are automatically run with default parameters. The option to estimate the tree in IDEA is particularly well suited to studies that include a large number of datasets and in which a good approximation to the true phylogeny is sufficient to address the particular questions of interest. If desired, trees previously generated outside of IDEA may be used instead. In a multi-dataset analysis, a separate phylogeny may be estimated or provided for each dataset. This is especially useful when the set of species analyzed is not the same for all datasets.

**Figure 2:**
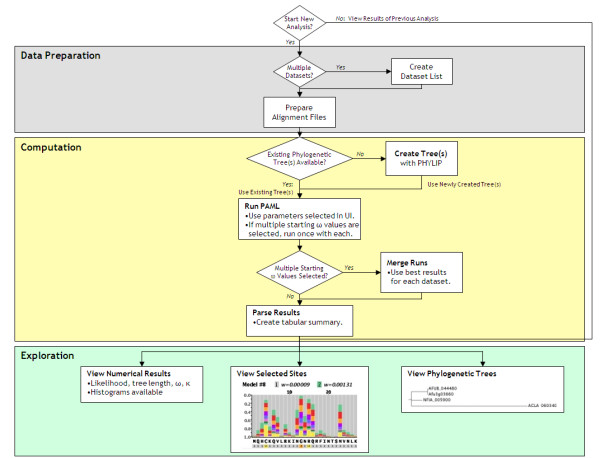
**The IDEA pipeline**. Flowchart of the four basic steps in an IDEA analysis: data preparation, phylogenetic tree acquisition, molecular evolution parameter estimates and result display and exploration. The course of the analysis is determined at four decision points: when choosing whether to launch the input or output GUI, when defining the nature of the data, when acquiring a tree and when compiling the results.

**Figure 3:**
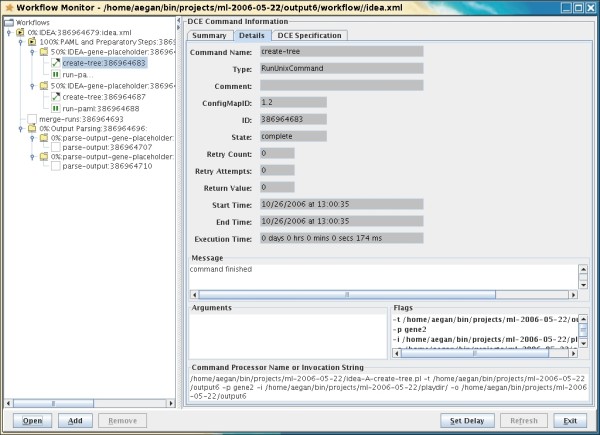
**Process monitor**. The Workflow Monitor allows the user to see which processes are running and peruse command output as it is generated. A hierarchy of jobs to be executed is displayed on the left. Clicking on a node on the left produces a detailed report on the execution status of that job or subset of jobs on the right. Information available includes start time, end time, execution host, percentage of jobs completed, execution time and command-line output.

Once the computational utility and parameters are specified, the IDEA pipeline processes each dataset (typically a set of orthologs) in four steps (Figure 2). The user may monitor the progress of an analysis by summoning the graphical monitor, a part of the Workflow process-management suite (Figure 3). Icons indicate which steps of the process are running, completed or waiting to start. The View Outand View Err buttons allow the user to see the complete command-line output of each process.

### Visualization

IDEA's input interface allows the user to run any *baseml *or *codeml *analysis. However, its output display is currently configured primarily for *codeml *analyses using site models or run in pairwise mode. IDEA's output GUI displays the results of *codeml *runs in an intuitive format that gives the user immediate access to relevant molecular evolution results. Its layout differs for pairwise analyses (involving only two sequences) and tree-based analyses (involving three or more sequences).

**Figure 4:**
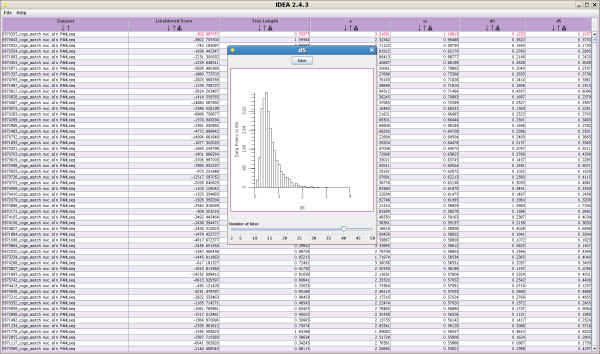
**Results display for a pairwise analysis**. The output display for a pairwise analysis consists of a data table. The following variables are displayed: dataset name, likelihood score, tree length, κ (the transition-to-transversion rate ratio), ω(*d*_*N*_/*d*_*S*_), *d*_*N *_and *d*_*S*_. Columns can be sorted (see ω) and the distribution of parameters across datasets visualized with a histogram. Genes with ω > 1 are displayed in red. The histogram indicates a modal *d*_*S *_value of ~0.5 (i.e., differences in ~50% of all synonymous sites) between the two *Plasmodium *species analyzed (see Results).

The output GUI's core element is a table listing the main parameter values estimated in the analysis. In the case of a pairwise analysis, the parameters displayed are likelihood score, tree length, κ (the transition-to-transversion rate ratio), *ω *(*d*_*N*_/*d*_*S*_), *d*_*N *_and *d*_*S *_(Figure 4). The table can be sorted based on any column and saved in tab-delimited text format for easy parsing. A histogram is available for each column of parameters estimated by *codeml*. The number of bins in the histogram can be adjusted interactively to fit the data.

**Figure 5:**
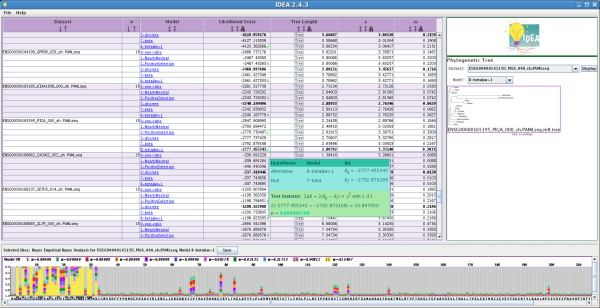
**Results display for analysis using more than two sequences**. The output display for a 'site models' *codeml *analysis with three or more sequences includes a sortable data table (columns: dataset name, number of sequences, model(s) of evolution used, likelihood score, tree length, *κ *and *ω*), a graphical depiction of a gene's phylogenetic tree (right panel) and a display of selective pressure along a gene (bottom panel). Results for an analysis of 104 datasets of 15 species each under six different models of evolution (NSsites = 0, 1, 2, 3, 7 and 8) are (partially) shown. Check marks next to likelihood scores for the *PIG-A *gene (third dataset from top; ENSG00000165195) indicate positive results for likelihood ratio tests (LRTs) on nested models 1-2 and 7-8; details of LRTs are available on mouse over (teal and green box). The selective pressure display (lower panel) features, for each amino acid residue, a stacked bar chart of the probabilities that the degree of selective constraint on that residue falls into each of several *ω *classes indicated by the color legend. While most residues in the PIG-A protein are extremely conserved (they fall with high probability in the gray class, which has the lowest *ω *value), there is strong evidence that the residues on the 5' end of protein are evolving under positive selection (yellow class, which corresponds to *ω *> 1)

The table displaying the results of analyses of three or more sequences shows, for each dataset, the following characteristics: number of sequences, model(s) of evolution (corresponding to *codeml*'s NSsites parameter), likelihood score, tree length, κ and *ω *(*d*_*N*_/*d*_*S*_). When multiple models of evolution have been run, the parameter estimates under each model are shown in a separate row. The row for the model with the highest likelihood in each dataset is shown in bold, and rows with *ω *≥ 1 are highlighted (Figure 5).

Two pairs of evolution models available in *codeml *(M1a and M2, as well as M7 and M8) are nested; that is, one is a specific case of the other, more general, model. Each of these nested pairs comprises evolution models that are identical except that the second allows some amino acid residues to evolve under positive selection (i.e., to have ω > 1) whereas the first does not. When a nested pair has been analyzed, the results table indicates the outcome of a likelihood ratio test performed to determine if there is statistical support for the hypothesis of the presence of positive selection during the evolution of the gene. This test is based on the p-value for the test statistic 2Δℓ (twice the difference in likelihood of the two nested models); this p-value is obtained using the PAML tool *chi2*. If the likelihood ratio test suggests positive selection, a check mark is displayed next to the model that allows positive selection; otherwise, an X is displayed. This feature enables the user to immediately pinpoint genes with evidence of positive selection. The details of a likelihood ratio test can be viewed by mousing over the check or X mark (green and teal panel in Figure 5).

To the right of the data table for a tree-based analysis is a graphical depiction of the phylogenetic tree for a given dataset under a given model. The lengths of branches in the tree are proportional to the evolutionary distance between nodes as estimated based on that model. The tree's topology can be quickly evaluated; this is particularly useful for analyses in which trees were first estimated in the IDEA pipeline.

Below the tree-based analysis data table is a scrolling display of ω values for each amino acid residue in the sequence alignment for a dataset selected in the main table. This colorful display, based on the *codeml *text output file "rst", features a stacked bar chart of the probabilities that each site evolves with an *ω *value in one of several ranges determined by *codeml *based on the data and the evolution model. Fast-evolving protein regions are immediately obvious from the bar chart (predominantly yellow region in the bottom panel of Figure 5). Individual amino acid residues with probability > 50% of being under positive selection are shown in reverse color (white letters on a black background).

## Discussion and conclusion

### Experimental results obtained using IDEA

We undertook several experiments both to benchmark IDEA and to illustrate its usefulness for the exploration of data.

First, to quantify the time-saving potential of IDEA's parallel processing feature, we performed a *codeml *analysis on 104 mammalian genes obtained using OrthoMaM [13] using a cluster of three 8-core, 3-GHz Intel Xeons with 16 GB of RAM each. This allowed 24 processes to execute simultaneously, with an average of 2 GB of RAM per processor. Each gene dataset had sequences for 15 species and was analyzed twice (once with each of two starting ω values) using *codeml*'s NSsites models 0, 1, 2, 3, 7 and 8. This analysis took 4 hours, 43 minutes, 52.505 seconds. In comparison, performing the same analysis on 10 of these genes on a single core of one of these machines took 9 hours, 42 minutes, 4.488 seconds, or about 21.3 times longer. The increase in average single-core run time per dataset between the parallel single-core analysis (58.2 min/dataset) and the cluster run (65.5 min/dataset) reflects the additional time required for task scheduling on multiple machines.

The performance of IDEA in pairwise run mode was tested using a set of 3,322 orthologous gene pairs from two *Plasmodium *species, *P. vivax *and *P. knowlesi*. Pairwise *codeml *analyses are much faster, allowing an analysis of many genes to run in a workable amount of time. Indeed, the 3,322 gene datasets were analyzed in 8 hours, 54 minutes, 15.257 seconds on a single core of one of our eight-core Intel Xeons. By contrast, using our cluster of three Xeons, performance deteriorated to 11 hours, 55 minutes, 25.345 seconds. This can be attributed to job scheduling overhead and demonstrates the importance of selecting an execution mode (local vs. distributed) to match the data. Our results suggest that, for pairwise analysis, IDEA can be most efficiently run on a single machine.

These analyses also present opportunities to demonstrate the usefulness of IDEA's interactive output display. The analysis of one of the genes in the 104 genes/15 species analysis, *PIG-A *(phosphatidylinositol N-acetylglucosaminyltransferase subunit A), and in particular the comparison of the two nested evolution models M7 and M8, suggests the presence of several positively selected sites, even though the overall estimated ω value for the gene, based on model M8, was a relatively low 0.3033 (Figure 5). The display of selective constraints along the gene (based on model M8; figure 5) clearly indicates that the sites under positive selection are concentrated in the first 30 amino acid residues. This type of signal is made quickly available to the user of IDEA, without requiring any additional data handling or parsing, and can subsequently be interpreted in the context of protein structure and function, cellular localization or other appropriate attributes of the gene in question.

The usefulness of IDEA for data exploration in pairwise analyses was investigated using genome-wide data from *Plasmodium vivax *and *P. knowlesi*, two closely related parasitic species, for which the primary mammalian hosts are humans and monkeys, respectively. Previous analyses of the three mitochondrial genes from these species showed a divergence in synonymous sites of only 1.9% [14], suggesting a very recent common ancestry between the two species. In order to determine whether the nuclear genome corroborated this result, an analysis was conducted on the set of 3,322 orthologous nuclear gene pairs for the two species used in the benchmarking experiment. A histogram of *d*_*S *_values obtained for the two species (Figure 4) shows a modal *d*_*S *_of approximately 50%, revealing a clear discordance between the mitochondrial and nuclear genomes and implying a much older divergence time between the two *Plasmodium *species than it was previously believed (J.C. Silva, in preparation).

The accuracy of IDEA's estimates was assessed using 5,445 single-copy ortholog sets in human, mouse and rat. Based on these genes, we calculated a median *d*_*S *_of 0.1969 between mouse and rat. Unlike *d*_*N *_or *ω*, the *d*_*S *_value is fairly independent of the genes included in the analysis and can therefore be compared across datasets. The value we obtained is consistent with previous estimates of 0.19 [15], 0.212 [16] and 0.163 [17]. Similarly, a median *d*_*S *_of 0.6078 for human and mouse and 0.62035 for human and rat are comparable to previous results [15,17,18].

### Avenues for further development

The modular organization of the IDEA pipeline greatly simplifies alterations to its back-end processes. Planned expansions of IDEA's capabilities include using PHYML [19] for phylogeny estimation and extending IDEA's output GUI to support a wider range of PAML analyses, including the graphic display of *baseml *results as well as those from branch and branch-site *codeml *analyses, which allow ω to vary across branches of a phylogeny as well as across sites in a gene. Other goals are to allow the user to launch any of the PAML analysis programs and to resume an aborted analysis mid-execution.

Despite the current existence of software with overlapping capabilities, such as HYPHY [20], PAML remains an extremely popular program to use for molecular evolution analyses. IDEA simplifies the process of running analyses using two of PAML's core components, *codeml *and *baseml*, and provides an intuitive graphical interface for the exploration of *codeml *results. The ability to launch and monitor analyses and to parse, view and explore results all in a single tool significantly expedites data handling and result acquisition. The ability to analyze multiple datasets in parallel can provide an improvement in run time of over an order of magnitude, and IDEA's ability to display the results of multiple dataset analyses in a single interactive table is unmatched. These advantages make IDEA ideally suited to large datasets such as those encountered during genome-wide molecular evolution analyses.

## Availability and requirements

IDEA is available at http://ideanalyses.sourceforge.net and is free to non-commercial users. Documentation and a discussion area are also available on SourceForge. System requirements include a Linux, Solaris or Mac OS X operating system and the installation of programs used by IDEA, including PAML and PHYLIP. Currently, either Condor or SGE is required if the ability to distribute jobs on a computing grid is desired; other job schedulers, including Torque/PBS, will be supported in an upcoming release.

## Authors' contributions

JCS and JMC conceived the project. JCS developed the initial computation pipeline. RS developed and tested IDEA. AM developed the Workflow suite. JC contributed the code for the selected sites display. JHB contributed code for drawing trees and interfacing with PHYLIP to create trees. RS and JCS wrote the paper.
